# On the Objects and Mutual Relations of the Medical Sciences: An Introductory Address Delivered at the Middlesex Hospital School of Medicine, at the Opening of the Winter Session

**Published:** 1839-04

**Authors:** 


Art. II. — *On the Objects and Mutual Relations of the Medical Sciences: an Introductory Address delivered at the Middlesex Hospital School of Medicine, October* 2, 1838, *at the opening of the Winter Session.* By Francis Leighton, m.d., Lecturer on Forensic Medicine at the Middlesex Hospital School of Medicine.—*London,* 1838. 8vo. pp. 51.

This elegant and judicious discourse, on which we have space only to make the briefest comment, may be regarded as a very pleasing indication not only of the lecturer's acquirements and his liberal views of science, but of the attainments and character of the pupils to whom such discourses are addressed. We observe with the sincerest pleasure such proofs of the respectability of any school; they afford a most grateful contrast to the wordy advertisements into which it was sometimes thought expedient, not many years ago, to dilate such addresses, as a foreshadowing of cheap and ready certificates.

